# Guidelines for Reporting Articles on Psychiatry and Heart rate variability (GRAPH): recommendations to advance research communication

**DOI:** 10.1038/tp.2016.73

**Published:** 2016-05-10

**Authors:** D S Quintana, G A Alvares, J A J Heathers

**Affiliations:** 1 Division of Mental Health and Addiction, NORMENT, KG Jebsen Centre for Psychosis Research, University of Oslo, Oslo University Hospital, Oslo, Norway; 2 Telethon Kids Institute, University of Western Australia, Perth, WA, Australia; 3 Cooperative Research Centre for Living with Autism (Autism CRC), Brisbane, QLD, Australia; 4 School of Psychology, University of Sydney, Sydney, NSW, Australia; 5 Department of Cardiology and Intensive Therapy, Poznań University of Medical Sciences, Poznań, Poland

## Abstract

The number of publications investigating heart rate variability (HRV) in psychiatry and the behavioral sciences has increased markedly in the last decade. In addition to the significant debates surrounding ideal methods to collect and interpret measures of HRV, standardized reporting of methodology in this field is lacking. Commonly cited recommendations were designed well before recent calls to improve research communication and reproducibility across disciplines. In an effort to standardize reporting, we propose the Guidelines for Reporting Articles on Psychiatry and Heart rate variability (GRAPH), a checklist with four domains: participant selection, interbeat interval collection, data preparation and HRV calculation. This paper provides an overview of these four domains and why their standardized reporting is necessary to suitably evaluate HRV research in psychiatry and related disciplines. Adherence to these communication guidelines will help expedite the translation of HRV research into a potential psychiatric biomarker by improving interpretation, reproducibility and future meta-analyses.

## Heart rate variability research in psychiatry

Heart rate variability (HRV) is the complex modification of the heart rate by the coordination of autonomic, respiratory, circulatory, endocrine and mechanical influences over time. Originally popularized as a research tool to detect fetal distress,^1^ and later to predict risk of mortality post-myocardial infarction using 24-h Holter recordings,^2, 3, 4, 5^ quantification of HRV has recently been more widely adopted to approximate autonomic control of the heart rate in the short term.^6, 7, 8^ The use of HRV as a transdiagnostic marker has a long research tradition in psychiatry^9^ that dovetails the recent push to establish neurobiological markers of psychiatric illness for improved nosology.^10^ Meta-analyses have established that individuals with a range of psychiatric disorders have reduced HRV, with the greatest reductions observed in psychotic disorders^11, 12, 13, 14^ (but see Stein *et al.*^15^ for situations where higher HRV is not necessarily better). The increased incidence of cardiovascular disease in psychiatric illnesses compared with healthy controls^16, 17, 18^ has also contributed to the increasing interest to better understand autonomic nervous system function in psychiatric illnesses. HRV has also been shown to covary with a range of psychological phenomena that are impaired in psychiatric illnesses such as social cognition^19, 20^ and executive function.^21, 22^ HRV is a central component of two prominent biobehavioral frameworks: the neurovisceral integration model, which highlights an inhibitory cortico-subcortical neural circuit to respond to environmental challenges^23^ and the Polyvagal theory, which adopts a phylogenetic approach.^24^ Both models emphasize how reduced HRV approximates a failure to inhibit maladaptive cardiac autonomic response to stress and perceived threats, whereas increased HRV promotes behavioral adaption and cognitive flexibility, all of which are inherent features to a number of psychiatric illnesses.

Considering the relationship between HRV and many core clinical features of psychiatric illness, the efficacy of novel treatments designed to increase HRV and, perhaps concordantly improve symptoms, is now being explored. HRV biofeedback has been shown to increase HRV by training resonance frequency breathing, which is typically 5.5 breaths per minute on average,^25^ but can vary from person to person.^26^ Early HRV biofeedback trials have demonstrated good tolerability and modest symptom improvements in anxiety, mood and substance-use disorders.^27, 28, 29, 30, 31^ Vagus nerve stimulation (VNS), which involves surgical implants of electrodes to the left vagus nerve, also increases HRV.^32, 33^ VNS has demonstrated effectiveness in treatment-resistant depression,^34, 35, 36, 37^ with the US Food and Drug Administration granting approval for such therapeutic use of VNS in 2005. Owing to the risks of surgery, however, VNS is only indicated for the most severe cases of depression. Non-invasive transcutaneous VNS that stimulates afferent vagus nerve fibers located in the ear^38^ has also demonstrated similar results,^39, 40^ which may open up such treatment to more individuals with depression.

Many HRV studies in psychiatry refer to a set of standards established almost two decades ago.^7^ While the general principles surrounding data collection, analysis and interpretation outlined in this report remain relatively unchanged (apart from novel HRV parameters^41^), coordinated efforts to improve research reporting and reproducibility have only recently emerged.^42, 43^ Given the absence of reporting standards specific to psychiatric research, it is therefore unsurprising that critical study details are inconsistently communicated.^14, 44^

## The drawbacks of inconsistent study reporting

Inconsistent descriptions of study methodology present a significant problem for the scientific community at large. First, a lack of methodological detail may hinder or delay peer-review. Almost half of a large sample of researchers (*n*=4037) described the peer-review process as ‘slow' or ‘very slow'.^45^ Appropriate reporting of HRV methods will reduce the need to request additional technical details, which then need to be gathered and structured by the original authors, benefitting both reviewers and editors. Standardized reporting of HRV methods will also benefit authors, assisting in the design of experiments and data collection.

Second, a lack of methodological detail hinders replication. A growing movement within the scientific community supports the standardization of reporting methodological details and providing transparent access to original data to improve the odds of replicability. Such large replication projects have described similar impediments because of a lack of methodological details in non-biological psychology,^43, 46, 47, 48^ neuroimaging^49, 50^ and drug discovery.^51^ Ostensibly, the goal of a methods section is the description of methodology such that findings may be replicated from written papers without clarification or reference to other sources. However, the reporting of methodological details required for replications is often inconsistent.

Third, unclear methodology presents inevitable and occasionally insoluble problems for performing meta-analyses; that is, the statistical combination of multiple studies to calculate a summary effect size for a given research question. Even combining effect sizes from two or three sets of data meaningfully increases statistical precision.^52^ However, the quality of data reporting in HRV research is mixed, if it is even available, and requests for additional data because of incomplete information often yield low response rates.^14, 44^ As large replication endeavors have often noted, retrospective requests for data or detailed protocols may be difficult to fulfill; for instance, data retention requirements for psychological or clinical data vary between institutions, the responsible students or staff may not be present at the time of the data request or data may be lost or corrupted because of older electronic data-storage systems. Moreover, the initial lead researcher may have no time for, or interest in, assisting with replication or meta-analyses. Researchers occasionally invoke the violation of privacy regulations, such as the Health Insurance Portability and Accountability Act, when declining data-sharing requests. Many methods have been developed that comply with Health Insurance Portability and Accountability Act-compliant de-identification.^53, 54, 55^ In cases where data cannot be appropriately de-identified (for example, participants all live in a specific geographical region), additional privacy safeguards, such as data-usage agreements, can be implemented.^56, 57^ To facilitate the sharing of data, consent processes can be updated to inform potential participants who de-identified study data may be shared.^56, 58^ Researchers may also associate direct replication with implicit criticism and may be uncomfortable with other laboratories pursuing their initial protocols as replication may miss the nuance of the original protocol.^59^ In short, insufficient reporting may slow, obstruct or even directly impede the calculation of meta-analytical effect sizes and related moderator analyses.

**Figure 1:**
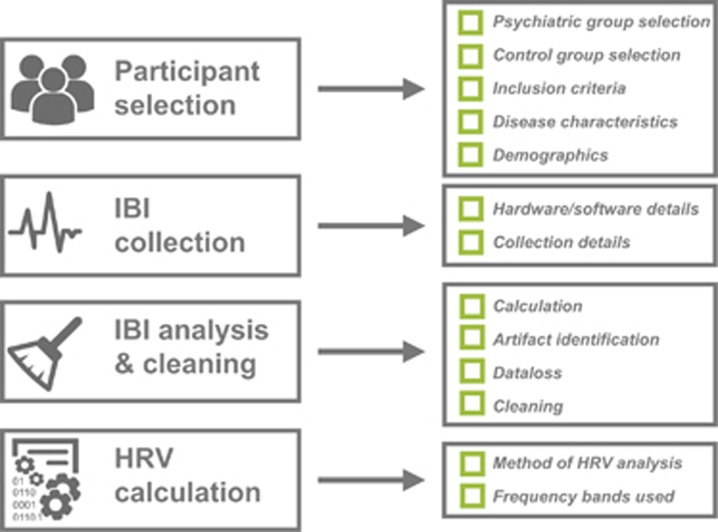
Guidelines for reporting articles on psychiatry and heart rate variability (GRAPH). A minimum set of criteria from which to design and report HRV studies in psychiatry. IBI, interbeat interval.

In consideration of these factors, we propose the Guidelines for Reporting Articles on Psychiatry and Heart rate variability—GRAPH. Research reported according to the 13-item GRAPH checklist (Figure 1 and Table 1; also see Supplementary Table 1 for a downloadable template) will help expedite translational research efforts by reducing the need for data and protocol requests for meta-analysis, replication and peer-review. Most importantly, the reader will have increased means to examine research in the field critically. We cover important considerations for HRV studies in psychiatry and biobehavioral research that include the following: selection of participants, interbeat interval (IBI) collection, data preparation and HRV calculation. Although we hasten to emphasize that we are not attempting to advocate a *standard* for HRV research—the breadth of research questions and methods renders this impractical—providing this information will help improve the interpretation of HRV research in psychiatry and related disciplines. Although not an exhaustive list of all the potential methodological considerations for the collection and analysis of HRV data, these guidelines are intended to provide a minimum set of criteria from which to design and report HRV studies in psychiatry.

## Participant selection

The selection and description of study participants is an integral, but oft-under-reported aspect of HRV research in psychiatry. Proper appraisal requires a minimum standard of information on study populations, particularly for case–control designs. When studies include a psychiatric population, for example, the method of diagnosis is an important detail considering the variability of classification accuracy. Different classification systems are available for diagnosis (the Diagnostic and Statistical Manual for Mental Disorders and the International Classification of Diseases). These diagnoses can be determined via structured clinical assessments administered by specialists and non-specialists. Indeed, inexperienced interviewers, such as graduate students, can have difficulties classifying psychiatric illness.^60, 61, 62^

Diagnoses can also be gathered via self-report. Simple self-reported diagnoses are the least accurate means of collecting diagnostic information; as many as half of patients are unaware or unable to correctly identify their diagnosis.^63^ Data from self-report questionnaires may show satisfactory agreement with structured clinical interviews and clinician diagnoses. However, they cannot replace clinical interviews for diagnosis, a point emphasized by the authors of many of these screening instruments.^64, 65, 66^ An additional confound is the large range of available self-report questionnaires, with variable validity, rendering comparisons between studies difficult. Data from participants with subclinical symptomology, particularly ‘high-trait' groups, based on these self-report questionnaires are still valuable, but such distinction needs to be explicit (for example, self-report questionnaire cutoffs). Disorder characteristics can also influence HRV. For instance, age of onset and illness severity are associated with HRV.^13, 67^ Finally, psychiatric comorbidities, which are common in psychiatric illness,^68^ also modify HRV in psychiatric populations.^69^

Healthy participants are often recruited to HRV studies to study behavioral or cognitive correlates, as a comparison with a clinical population, or a combination of both these goals. Bearing in mind the well-described association between mental illness and HRV, adequate descriptions (as detailed above) of how the absence of the condition was determined in controls are important. This is not only relevant in studies that compare HRV between a psychiatric population and controls but also studies that exclusively report the recruitment of healthy controls. Relatedly, the source of the healthy comparison group is also relevant. Many studies recruit ‘hypernormal' controls (also referred to as ‘well' controls) who are not representative of the general population.^70, 71^ Although it is ideal to recruit participants from the same sampled population as the clinical group, this may not always be possible or practical because of cost and time considerations (but see Schechter and Lebovitch^72^). Specific information about where control groups were selected from can provide a more accurate assessment of whether differences between groups may be exaggerated by potential control group population biases (for example, socioeconomic status and race).

Irrespective of the psychiatric status, reporting of pre-specified participant exclusion and inclusion criteria requires adequate description. Research indicates that demographic attributes, such as gender,^73^ physical activity levels^74, 75^ and habitual levels of alcohol,^11, 76^ and nicotine intake^77^ influence HRV. Age is of particular importance as it is inversely related to HRV^78, 79^ and patient groups are often older compared with the younger university/college students usually recruited to healthy sample studies. The occurrence of physiological conditions^80, 81^ and ectopic beats also increases with age.^82, 83^ In addition, physical health conditions such as cardiovascular,^84, 85, 86^ metabolic^87^ and renal diseases^88, 89, 90^ can have an impact on HRV and occur at increased frequency in psychiatric conditions. Relatedly, cardiovascular and psychotropic medications (especially, tricyclic antidepressants) also have an appreciable impact on HRV.^13, 14, 91^ Assessing for these factors is particularly important when comparing clinical and nonclinical groups, as they can often differ on many of these domains but may not be standardly assessed or reported.

## IBI collection

### Hardware

Interbeat interval data have traditionally been collected via electrocardiogram (ECG) or photopletysmography. These methods still represent the bulk of recordings, although more recent technologies are available or in development, such as smartphone-enabled optical pulse sensors,^92^ webcam video,^93^ ultrasounds to index fetal heart period,^94^ microwave radar,^95^ cushion-mounted ballistocardiogram^96^ and toilet seats.^97^ To assist interpretation and reproducibility, research should communicate details of the device (for example, manufacturer and model) along with the software used for IBI extraction or analysis. If the device is commercially unavailable, the researcher should provide more detailed information, including but not limited to the analog front-end, microcontroller unit, peripherals, electrodes and so on. Likewise, any data concerning explicit validation against existing measurement devices should be included. Many sports watch-monitor manufacturers offer models with sufficient sampling rates to calculate HRV (for example, Polar and Suunto). However, researchers do not have access to metrics about how these devices are identifying or correcting errors, consequently making the decision on the data quality sufficient for retention in analyses difficult. Error identification might be possible to some degree from RR intervals, but these devices do not provide the ECG trace, which can be used to better identify cardiac dysrhythmia. Thus, using these devices to investigate a condition or population characterized by above-average error or ectopy may be problematic.^98^

Recent advances in technology along with the rise of the ‘quantified self' movement,^99^ where people track and monitor their own biometric data, have converged to create a new category of consumer devices purported to measure HRV. However, these devices are generally not formally validated against an ECG for accuracy. Moreover, these devices often (a) report a proprietary metric rather than a standard metric, (b) do not provide access to raw data and (c) do not offer technical details of correction methods (if any are present). Researchers should consider their own investigations into the validity of novel devices to determine their accuracy in the population of interest. Without a method of checking the original sinus rhythm there is no way to determine the accuracy of potential errors in a beat-to-beat series. That is, a proprietary device may return accurate beat information, but other device and individual patient factors may go undetected. For instance, vascular insufficiency may interfere with the collection of photopletysmography signals, and cardiac arrhythmia may interfere with the collection of ECG signals, both of which may be impossible to determine without inspection of the raw data.

### Sampling rate

**Figure 2:**
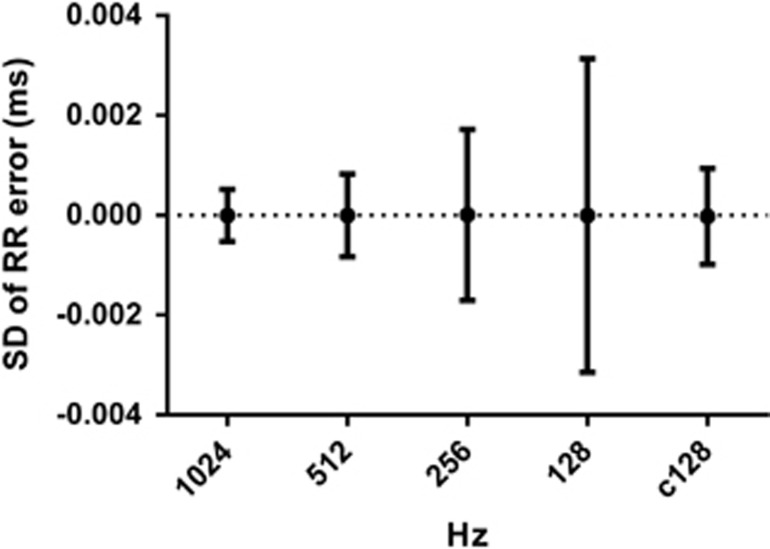
Reconstruction of interbeat interval (IBI) signal. s.d. of errors compared with a natively sampled 2048-Hz signal in a single 15-min recording where all IBIs are identifiable; a simple quadratic correction to the peaks of the 128-Hz signal (c128) results in comparative accuracy to the natively sampled 512-Hz signal.

While the electrocardiographic P-wave is the direct representation of sinoatrial (SA) depolarization, and therefore the closest indication of the initiation of the cardiac cycle, the R-wave (which corresponds to ventricular depolarization) is used for convenience. As temporal accuracy is important to calculate the variance of a time series successfully, previous guidelines provided a minimum desirable sampling rate,^7^ with 500 Hz being the recommended threshold to accurately identify native fiducial points.^100, 101^ Having said that, 250 Hz may also be adequate when collecting data from healthy adults.^102^ It is still possible that Holter legacy data, which was typically recorded at 128 Hz, may be below this minimum. However, early data recorded at 128 Hz may still provide useful information if the error subsequent to the slower sample rate is recognized. Altogether, this consideration is only occasionally relevant to contemporary research, as hardware standards are often well in excess of minimum sampling rates—commercial devices are readily available with native sampling rates of 1–8 kHz. Moreover, it is clear that R-wave fiducial points can be easily reconstructed from lower-sampled signals as a 128-Hz signal contains enough data to improve the information in the signal. For instance, the reconstruction of R-waves even with a simple quadratic correction allows 128-Hz data to show equivalent accuracy to 512 Hz (Figure 2). As a consequence, research should state the native sampling rate of the hardware utilized, along with any details of signal reconstruction (if applicable).

### Time factors of recording

Whereas HRV has historically been calculated using 24-hour Holter monitoring—which offers superior prediction of future cardiovascular disease mortality^103^ and the opportunity to evaluate longer-term circadian HRV differences—the majority of HRV data within psychiatry and the behavioral sciences have been calculated using short-term recordings (durations between 1 and 5 min). The Task Force statement suggests a minimum of 60-s continuous recording to quantify high-frequency (HF) HRV,^7^ with recent work showing reasonable agreement between ultrashort-term HRV measures (<60 s) and 5-min periods.^104^ Two other recommendations for time periods are common (although neither appears to be strictly analytical): (a) 5 min of baseline recording, which is an overwhelmingly popular standard for most frequency domain HRV recordings,^105^ and likely a vestigial standard leftover from the original processing limits of the PDP-8 minicomputers originally used to collect ECG during the 1960s; and (b) 10 cycles of the lowest frequency of interest, or at least 250 s of recording in a standard frequency domain analysis reporting a low-frequency (LF) band with a lower bound of 0.04 Hz.

A related issue for consideration is acclimatization to the recording environment. Often this is accomplished by using an analysis period that begins subsequent to the start of recording, an approach used by ourselves^19, 67, 92^ and others.^106, 107, 108^ Acclimatization can help reduce any HRV changes because of posture, which may take time to adjust if the participant has just assumed a supine or seated position.^109, 110, 111^ Acclimatization may also reduce confounds subsequent to test anxiety in psychiatric populations. Considering the impact of attentive states on respiratory frequency,^112^ which subsequently influences the ECG recording,^113^ the beginning of the recording period should not be announced. As the comparison of HRV between different time periods is also problematic,^114^ the longer period should be reduced to match the shorter period.

### Posture and procedures

Posture has a well-characterized effect on autonomic outflow, which is proportional to the shift of the body axis because of the primarily sympathetic response to venous pooling and consequent involvement of the baroreflex. The use of graded tilt—whereby an immobile participant's posture is increased in progressive increments from supine to upright—reveals a strong curvilinear relationship between posture and HRV.^109, 115^ Consequently, direct equivalence between supine, seated and standing recording is not warranted, given these differences and wider dispersion of HRV during supine recordings compared with upright.

The instructions given for a task are also an important consideration. In some cases,^116, 117, 118^ researchers administer a low-demand cognitive ‘Vanilla' Task,^119^ which may have some relevance for psychiatric populations that have difficulty sitting still or experience stress under experimental ‘resting-state' conditions. Instructions with attentional demands may also modulate HRV, primarily because of changes in respiratory depth and frequency. Regardless, the instructions given to the participant should be stated. Some researchers have also attempted to blind participants to the purpose of the equipment that measures respiration to encourage true spontaneous breathing. For instance, Vlemincx *et al.*^112^ explained to participants that respiratory sensors built into a garment were collecting information on muscle tension. Thus, instructions given to participants for baseline recordings should be noted. Considering the influence of circadian rhythms and digestion on HRV,^120, 121, 122, 123^ time of day and time since last meal should also be noted and standardized where possible in short-term HRV recordings, especially for designs incorporating repeated recordings over time.

### Other signals

The obvious ancillary recording for the measurement of HRV is respiration, due to its direct influence on HF HRV.^124, 125, 126^ However, the importance of monitoring respiration under resting conditions is not settled. Proponents for respiratory monitoring maintain that respiration should be controlled, as respiratory parameters may modify the relationship between HF HRV and cardiac vagal modulation.^127^ Others argue that this is not necessary,^128^ as these respiratory and HR oscillations have the same origin.^129^ Regardless, respiration is almost exclusively the source HF HRV (with some modest contributions from other sources, such chest compression^130^), and respiratory data provide information that directly affects the cardiac cycle, such as respiratory frequency and depth. Erratic sinus arrhythmia (see more information below) may also contribute to the HF band despite its non-respiratory origin.^15^ Indirect measures of respiration can also be calculated from ECG^131, 132, 133^ and photoplethysmographic^134^ signals. The potential to compute respiration directly from a record of cardiac intervals without the need for monitoring via impedance, belt or mask is appealing, considering its simplicity. In specific situations, respiration may be of interest to researchers investigating populations that happen to breathe slowly, such as athletes^74^ or meditators.^135^ On the other end of the spectrum, some patient populations,^136, 137^ and children^138^ breathe at a faster rate. There is evidence to suggest that periodic and sudden fluctuations in the respiratory rate are both a source of altered HRV variability over time and are directly mediated by experimental task demands.^112^ The presence and frequency of sighs, which are reported to be increased in psychiatric illnesses,^139, 140^ should also be monitored as these produce large deviations from typical respiratory length and depth.^141^ HRV is also dependent on HR in an inverse nonlinear manner.^142, 143^ A mathematical correction for this dependency has been proposed to improve HRV reproducibility.^144^ Finally, using exercise as a stress task will also increase the respiratory rate.^145^

## Data cleaning and analysis

### IBI calculation

Identifying IBIs is computationally straightforward; the Pan-Tompkins algorithm,^146^ where the raw ECG signal is bandpassed, differentiated, squared, integrated and smoothed to isolate R-waves, was developed over 30 years ago and remains a common and effective processing method. After peak detection is achieved, most frequency domain analysis methods resample the RR series into an evenly sampled time series (typically between 1 and 10 Hz). Different resampling rates may affect equivalent frequency transforms; however, this usually is not profound as long as researchers satisfy the Nyquist criterion.^147^

Several sources of artifact that may affect the frequency bands of interest are common.^148^ The main sources of contamination include powerline interference (at 50 or 60Hz depending on the nature of local AC power), muscle contraction or movement artifacts, and baseline drift. The occurrence of one or more of these in any given data set is almost certain, but may be highly variable; therefore, filtering of data for artifacts should be clearly noted in manuscripts along with whether beat detection was visually inspected as some systems (predominately clinically oriented suites) do not provide this facility. To aid future analysis, the raw signal should be recorded without online filtering during data acquisition, if possible.

### Non-sinus beat/arrhythmia identification

**Figure 3:**
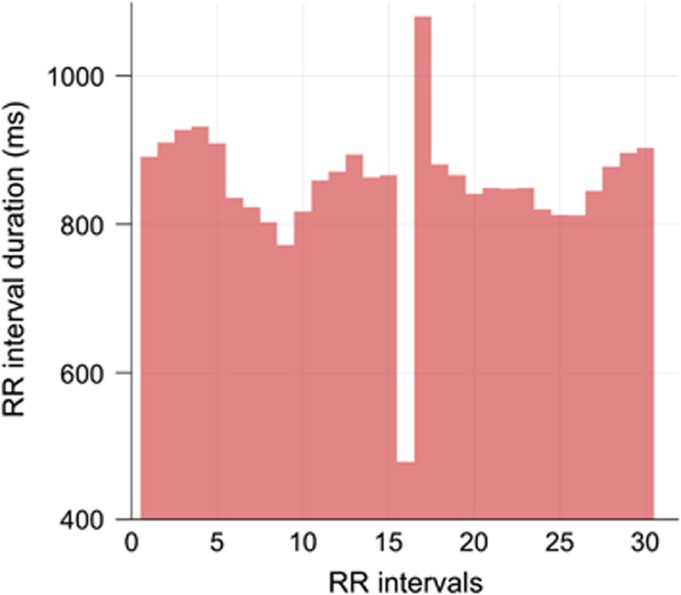
A tachogram illustrating an ectopic beat in an R–R interval time series. An ectopic beat appears as a short R–R interval (480 ms; R–R interval 16) followed by a compensatory pause (1080 ms; R–R interval 17).

A central premise of HRV is that IBIs approximate SA node-firing patterns, otherwise known as normal sinus rhythm. Accordingly, a fiducial point that does not originate from the SA node (that is, a non-sinus beat) does not represent the autonomic nervous system input to the SA node. Two common sources of non-sinus beats are the atria and ventricles, which can prompt an atrial premature contraction and ventricular premature contraction (VPC), respectively. As these beats are premature, they lead to a short R–R interval followed by a long compensatory R–R interval (Figure 3). Pathological causes of ectopy include electrolyte abnormalities, ischemia and cardiomyopathy. Ventricular arrhythmias can also be generated from an unmasked ectopic focus in populations with very low HR, such as athletes.^149^ More benign causes of ectopy in healthy populations include caffeine or nicotine,^150, 151^ which is one reason most researchers ask participants to refrain from such intake before IBI collection. To our knowledge, research is yet to examine the impact of caffeine withdrawal on HRV, which requires future investigation. However, double-blind experiments suggest that abstinence in those that regularly consume caffeine has no effect on heart rate.^152^

Although even one misidentified beat can have a considerable influence on HRV calculation,^153^ there is no consensus or absolute criteria for defining atrial premature contraction or VPCs via algorithm. Accordingly, manual inspection of the ECG trace is needed for non-sinus beat identification. Many software packages attempt to make their own corrections from R–R intervals, not from identifying incorrect ECG signals. Atrial premature contractions and VPCs are relatively common; a few percent in a healthy population,^82, 83^ a higher percentage in an athletic population^154, 155^ and higher still in some cardiac conditions.^156^ However, many papers do not seem to have any corrections or removals at all or omit these details entirely.

Correcting these artifacts assumes only that the directly affected beats are problematic. However, a further concern exists if VPCs are the source of heart rate turbulence, in which a non-sinus beat causes subsequent short-term R–R changes.^157^ In healthy people, the compensatory pause after a VPC is followed by brief R–R interval acceleration, and then R–R interval deceleration. This pattern is not observed in high-risk patients, with decreased deceleration speed (that is, turbulence slope) shown to be a powerful predictor of mortality.^157, 158^ It is thought that heart rate turbulence has a baroreflex origin,^159^ whereby a VPC causes a brief drop in blood pressure, leading to baroreceptor inhibition of vagal input, which leads to a brief increase in heart rate. Careful inspection of the ECG signal can help both identify ectopy, as this can occur without artifact, and any subsequent heart rate turbulence. Visual inspection can also help identify artifacts that can be missed with commonly used filter thresholds. For example, people with extremely high or low HRV can have normal sinus beats misidentified as ectopic beats. Thresholds can also be adapted depending on the population.

Erratic sinus arrhythmia is a less well-recognized form of persistent arrhythmia that differs from ectopy in that heart beats appear to have normal electrocardiographic morphology (that is, they have sinus origin) but display short-term variation that is non-respiratory in origin.^15, 160^ Although erratic sinus arrhythmia has been strongly associated with serious cardiovascular illness, predicting post-infarction survival,^161^ this does not preclude it from occurring in other healthy or patient populations. An inspection of the Poincaré plot (a scatterplot where each point represents two consecutive heart periods) for regularity may be sufficient to reveal the presence of erratic sinus arrhythmia,^160, 162^ and should be performed on IBI series with inexplicably high HRV. Consequently, how beat inspection was performed, the percentage of beats identified per participant and any exclusions because of abnormal sinus rhythm should be explicitly stated.

### IBI data loss

There are many reasons for discarding data apart from persistent arrhythmia. These include hardware/software errors, wire loss, poor electrode contact, extraneous magnetic or line noise, excessive movement artifacts, EMG artifacts, experimenter error, file corruption, physical loss and accidental duplication. ECG signal characteristics, such as wide QRS complexes or tall t-waves, can also contribute to peak detection difficulties. These errors also influence how the viability of the experimental model is confirmed. For instance, movement-based tasks may destroy inappropriately filtered signals or experiments may use incorrect electrode sites or fail to manage data collection properly.

Data loss as a difference between groups is also concerning. For instance, a stress induction would conceivably cause more errors in a psychiatric sample compared with a control group. This leaves the investigator with a task by outcome interaction, which needs to be accounted for before normal task effects. The percentage of beats removed from the sample has a direct impact on accuracy, thus reporting that this percentage can be instructive. Ideally, the percentage of removed beats should be equivalent between groups to ensure that differences are because of the autonomic nervous system rather than artifact. Some methods preserve accuracy with data loss well; however, all methods perform badly when long sections are lost.^163^ This frequently happens with photopletysmography recordings because movement artifacts destroy adaptive filters, which often require 5–6 s to ‘re-adapt'. Five percent is the commonly stated threshold for R-peak data loss to render a series unusable (for example, Heathers *et al.*^164^), which to our knowledge has no empirical basis. However this, again, is a convention instead of an analytic barrier; therefore, the threshold used should be noted.

### IBI cleaning

When adjusting for errors, as above, the beat replacement method can directly affect measurement outcome. Just removing the beat is problematic, especially for shorter recordings and frequency domain calculations.^153^ Linear correction and cubic spline interpolation are acceptable solutions, but both potentially introduce errors (for a review of beat replacement techniques, see Peltola^165^). Time-domain methods, which are less sophisticated, also have the benefit that errors can simply be removed proportionally. Multiple consecutive errors cannot be handled the same way as single errors. If the researcher expects frequencies of up to 0.4 Hz in signals, then the loss of several beats in a row requires a different approach, such as discarding the sample entirely. However, work frequently combines and compares time and frequency domain methods of HRV. Consequently, a single correction method for replacement should be used before smoothing or decimation.

## HRV calculation

### Method of analysis used

There are more than 70 published metrics for calculating HRV.^166, 167^ Methods may be time domain (such as the s.d. of the NN interval), frequency domain (such as the Fast Fourier Transform), time–frequency domain (such as the continuous wavelet transform) or ‘nonlinear' methods, many of which are not strictly nonlinear (for example, correlation dimension, detrended fluctuation analysis, approximate entropy and sample entropy). Several direct equivalencies exist between these methods.^168, 169^ Frequency domain analysis is typically computed by fast Fourier transform or autoregressive techniques, which are almost equivalent for the HF band (*r*=0.96 Hayano *et al.*^170^). Other techniques include the Lomb–Scargle periodogram^171, 172^ and smoothed pseudo Wigner–Ville distribution.^173^

Although these methods may be similar, they should not be assumed directly equivalent under all circumstances.^174^ The resolution of a peak frequency in particular may be different between methods because of the smoothness of the power spectral density.^175^ All analytical results are subjected to a series of methodological assumptions (for example, windowing method, window length, overlap and frequency bands in frequency domain analysis). Therefore, the explanation of the analytic method chosen should be stated in enough detail such that a competent external researcher could reproduce the analysis with sole access to the manuscript. However, the exact details that this includes will differ with each method. As s.d. of the NN interval and power spectral density analysis (HF and LF powers) are the best characterized HRV metrics in terms of clinical use^41^ and historically the most commonly used metrics,^176^ continued reporting of these measures will aid replication and meta-analysis. If novel HRV methods are used, researchers should follow recent recommendations from the European Society of Cardiology, who suggest that novel methods should be reported in tandem with traditional HRV measures.^41^

### Selection and interpretation of frequency bands

Frequency domain analysis of IBI data is computationally straightforward. Whereas there are recommendations for bands to use in adults (LF, 0.04–0.15 Hz; HF, 0.15–0.4 Hz), children and infants (LF, 0.04–0.24 Hz; HF, 0.24–1.04 Hz) at rest because of spontaneous breathing rates, these are not necessarily important in other circumstances. For instance, some athletes have respiratory rates sufficiently slow as to interfere with the traditional interpretation of the measured HF band.^74^ Population characteristics should be considered when bands are chosen, either by looking at past research or by calculating respiratory rates of the data in question.

Disagreements over the teleology and mechanics of HF are minimal at present (but see Billman^176^), especially compared with disagreements over the nature of LF and the ratio between the bands LF/HF.^105, 177, 178^ For instance, LF is variously reported as a measure of ‘sympathetic activation', a component of activity in ‘sympathovagal balance' or a measure of sympathetic nervous system activity instead of activity of the baroreflex in response to vasomotor tone.^105, 179^ Subsequently, the Task Force paper^7^ requests reporting and analysis of raw LF and HF powers in addition to additional interpretation of those bands. Likewise, the parameters of those bands and their putative interpretation should be declared. These bands should also always be reported in standard format (that is, as LF and HF power in ms^2^/Hz) before further calculation. Finally, it is important to not overextrapolate short-term experimental recordings against established 24-h HRV findings.

## Additional considerations for reporting HRV studies

### Data and analysis script archiving

Development of new signal analytic techniques, confirmation of existing findings and meta-analyses rely on access not just to both existing results and the raw data from those results. Instead of relying on effect size comparison between studies with different methodologies for meta-analysis, pooling individual data points using mega-analysis would help adjust for within-subject differences in methodology.^180^ Mega-analyses have already been applied to other areas of biobehavioral research.^181, 182, 183^ The largest HRV meta-analysis in psychiatry to date, which included 170 studies, suggested that only 20–25% of observed heterogeneity was because of sampling error.^14^ This indicates that other factors, such as HRV calculation methodology, are contributing to heterogeneity in effect sizes. Moreover, mega-analyses can determine the degree to which differences in methodology contribute to heterogeneity compared with other heterogeneity sources.^184^ However, given the complexities surrounding data sharing,^185^ such as the precarious balance between openness and privacy, we feel that this cannot be included as a strict recommendation to publication. Nevertheless, the increasing movement from many publishers toward open-access sharing of data as an essential publishing requirement may mean that many of these heterogeneity issues could be addressed in future analyses of pooled individual data. Finally, the availability of the precise details of statistical analysis (that is, analysis scripts) may also improve reproducibility of research, as it offers the reader and reviewers the opportunity to closely examine how results were generated.

## Conclusion

The discipline of ‘meta-research' is a relatively recent proposal to accelerate the translation of scientific research by improving research methods, reporting, reproducibility and evaluation.^42^ HRV research in psychiatry tends to under-report important methodological details required for critical evaluation. For this reason, we have designed guidelines that consider important details related to this research to streamline reporting. These are not additional requirements for analysis, rather are criteria by which decisions that are made in every paper by necessity. The requested information already exists, in every paper, regardless of whether or not it represents a conscious decision by the author or has been omitted for the sake of brevity in the publishing process. We have summarized these guidelines in a 13-item checklist (Table 1 and Supplementary Table 1) and expect that adherence to these guidelines will improve reproducibility, expand the ability to perform meta-analyses, improve critical evaluation and expedite the peer-review process. Moreover, these guidelines will enhance the clarity of HRV research in psychiatry. Consideration of these guidelines at early stages of project planning will also aid study design. We make no attempt to recommend how HRV studies in psychiatry *ought* to be conducted because of sheer impracticality. The central question when assessing a methods section is to determine how the study reached its conclusions; therefore, the singular purpose of these guidelines is to facilitate the clear communication of research findings to accelerate the translation of psychiatric research.

## Figures and Tables

**Table 1:** GRAPH checklist items

*Topic*	*Number*	*Checklist item*
*Selection of participants*		
Psychiatric group selection	1a	Psychiatric group recruitment details and illness assessment methods
Control group selection	1b	Control group recruitment details and methods to rule out psychiatric illness
Inclusion criteria	1c	Description of inclusion criteria (for example, absence of physical health conditions)
Disease characteristics	1d	Description of disease duration, severity, psychiatric comorbidities and medication status
Demographics	1e	Details on age, gender distribution, physical activity level, alcohol intake and nicotine intake

*IBI collection*		
Hardware/software details	2a	Brand and electrode configuration (if applicable)
IBI collection details	2b	Raw sampling rate, length of data collection, time of day, filtering, participant posture and instructions

*Data analysis and cleaning*		
IBI calculation	3a	IBI calculation and resampling methods
IBI artifact identification	3b	IBI artifact identification method (for example, algorithm, manual inspection)
IBI data loss	3c	Reasons for loss (for example, persistent ectopy, equipment failure)
IBI cleaning	3d	Artifact cleaning methods and the percentage of beats were corrected

*HRV calculation*		
Method of analysis used	4a	Metrics used and the software/script used for HRV calculation, log transformation (if applicable)
Frequency bands used	4b	Specification of frequency bands and how they were interpreted

Abbreviations: GRAPH, Guidelines for Reporting Articles on Psychiatry and Heart rate variability; HRV, heart rate variability; IBI, interbeat interval.
