# Supplementary material for: Guidelines for Reporting Articles on Psychiatry and Heart rate variability (GRAPH): recommendations to advance research communication
Source: Transl Psychiatry. 2016 May 10;6(5):e803–. doi: 10.1038/tp.2016.73 (PMC5070064; doi:10.1038/tp.2016.73)
Supplement: Supplementary Table 1 [file tp201673x1.pdf]

Guidelines for reporting articles on psychiatry and heart rate variability (GRAPH) checklist items

Topic	Item number	Checklist item	Reported on page number
**Selection of participants**			
Clinical group recruitment and selection	1a	Psychiatric group recruitment details and illness assessment methods.	
Control group recruitment and selection	1b	Control group recruitment details and methods to rule out psychiatric illness.	
Inclusion criteria	1c	Description of inclusion criteria (e.g., absence of physical health conditions).	
Disease characteristics	1d	Description of disease duration, severity, psychiatric comorbidities, and medication status.	
Demographics	1e	Details on age, gender distribution, physical activity level, alcohol intake, and nicotine intake.	
**IBI collection**			
Hardware / software details	2a	Brand, electrode configuration (if applicable)	
IBI collection details	2b	Raw sampling rate, length of data collection, time of day, filtering, participant posture, and instructions.	
**IBI analysis and cleaning**			
IBI calculation	3a	IBI calculation and resampling methods.	
IBI artifact identification	3b	IBI artifact identification method (e.g., algorithm, manual inspection).	
IBI data loss	3c	Reasons for loss (e.g., persistent ectopy, equipment failure)	
IBI cleaning	3d	Artifact cleaning methods and the percentage of beats were corrected.	
**HRV calculation**			
Method of analysis used	4a	Metrics used and the software/script used for HRV calculation, log transformation (if applicable)	
Frequency bands used	4b	Specification of bands and how they were interpreted.	
